# Potentially Inappropriate Medications and Potential Prescribing Omissions in Elderly Patients Receiving Post-Acute and Long-Term Care: Application of Screening Tool of Older People’s Prescriptions/Screening Tool to Alert to Right Treatment Criteria

**DOI:** 10.3389/fphar.2021.747523

**Published:** 2021-10-19

**Authors:** Catarina Candeias, Jorge Gama, Márcio Rodrigues, Amílcar Falcão, Gilberto Alves

**Affiliations:** ^1^ CICS-UBI–Health Sciences Research Centre, University of Beira Interior, Covilhã, Portugal; ^2^ UMP–Union of Portuguese Mercies, Lisboa, Portugal; ^3^ CMA-UBI–Centre of Mathematics and Applications, University of Beira Interior, Covilhã, Portugal; ^4^ ESALD-IPCB–Dr. Lopes Dias School of Health, Polytechnic Institute of Castelo Branco, Castelo Branco, Portugal; ^5^ UDI-IPG–Research Unit for Inland Development, Polytechnic Institute of Guarda, Guarda, Portugal; ^6^ CIBIT–Coimbra Institute for Biomedical Imaging and Translational Research, University of Coimbra, Coimbra, Portugal; ^7^ Laboratory of Pharmacology, Faculty of Pharmacy, University of Coimbra, Coimbra, Portugal; ^8^ UFBI–Pharmacovigilance Unit of Beira Interior, University of Beira Interior, Covilhã, Portugal

**Keywords:** STOPP criteria, START criteria, inappropriate prescribing, prescribing omissions, elderly, Portuguese patients

## Abstract

**Background:** Screening Tool of Older People’s Prescriptions (STOPP) and Screening Tool to Alert to Right Treatment (START) criteria have been used to detect potentially inappropriate medications (PIMs) and potential prescribing omissions (PPOs). These criteria were applied to geriatric Portuguese patients receiving post-acute and long-term care to assess the prevalence and predictors of PIMs and PPOs.

**Methods:** An observational, retrospective, cross-sectional and multicenter study was performed in 161 patients (aged ≥65 years) from eight Units for Integrated Continuous Care.

**Results:** In these studied patients (mean age: 81.6, 64% female, median number of medications: 9) PIMs were detected in 85.1% and PPOs in 81.4% of patients. While PIMs mainly involved the central nervous system and psychotropic drugs (66.5%), PPOs were mostly related to musculoskeletal system (55.3%) and cardiovascular (39.8%) system. A subsequent analysis with logistic regression found the female gender, the hospital provenience, and the number of medications as predictors of PIMs. Predictors of PPOs were the Charlson Comorbidity Index and history of recent fractures.

**Conclusion:** PIMs and PPOs were highly prevalent in the studied patients receiving post-acute and long-term care in Units for Integrated Continuous Care. Therefore, STOPP/START criteria might be an effective tool for improving prescribing quality and clinical outcomes in these frail elderly patients.

## Introduction

Potentially inappropriate prescribing refers either to 1) potentially inappropriate medications (PIMs), the use of drugs where no clear clinical indication exists (*overprescribing*) or the use of an indicated drug where the risk outweighs the benefit or when a safer or more effective alternative is available (*misprescribing*) or 2) potential prescribing omissions (PPOs), not prescribing a beneficial medicine for which there is a clear clinical indication (*underprescribing*) (O'Mahony and Gallagher, 2008; O'Connor et al., 2012; Moriarty et al., 2015). In older people, this subject has been increasingly explored because of the relationship between potentially inappropriate prescribing and negative clinical outcomes, namely the occurrence of adverse drug reactions (ADRs) (Lindley et al., 1992; Hedna et al., 2015), risk of hospitalization, hospital readmission, lower quality of life, and even mortality (Akazawa et al., 2010; Dedhiya et al., 2010; Brown et al., 2014; Cahir et al., 2014; Thomas et al., 2020). This may be related to polypharmacy, which has been identified as a determinant factor for potentially inappropriate prescribing (Akazawa et al., 2010; Cahir et al., 2010; Bradley et al., 2012). Another concern is the cost, since the total expenditure on potentially inappropriate prescribing has been reported to be 9% of the global expenditure on pharmaceuticals in people aged 70 or over (Cahir et al., 2010). Moreover, in PIM users it was found an increase of 33% in healthcare medical costs comparatively with nonusers (Akazawa et al., 2010). Besides, it is also important to consider the potential impact of aging in drug elimination, because aging involves progressive impairments in the functional reserve of multiple organs such as liver and kidneys (Thomas, 2020). Considering that the number of people aged 65 years or over is projected to double, from 703 million to 1.5 billion, between 2019 and 2050, reaching a proportion of 16% worldwide (United Nations, D.o.E.a.S.A., Population Division, 2019) the high prevalence of PIMs in the elderly (Akazawa et al., 2010; Brown et al., 2014; Onder et al., 2014) is a current problem that will likely to be even worse in the future in this age group. Therefore, potentially inappropriate prescribing is a major concern that claims for measures that allow the detecting and reducing of its occurrence.

In order to improve prescribing, screening tools based on explicit criteria have been extensively used, being the earliest the Beers list (Beers et al., 1991), which was mainly applicable in the United States of America and has been updated in 2019 (American Geriatrics Society Beers Criteria® Update Expert Panel, 2019). Although this list was undoubtedly important to the advances in the study of PIMs, the criteria used could not be easily applied in European countries. Therefore, in the last decade, a European-based tool was also developed to detect PIMs and PPOs, respectively: 1) the Screening Tool of Older People’s Prescriptions (STOPP); and 2) Screening Tool to Alert to Right Treatment (START) (Gallagher et al., 2008; O'Mahony et al., 2015). The STOPP and START criteria consist of a list of PIMs and a list of PPOs, respectively, which complement each other. STOPP criteria can play an important role in reducing PIMs rates (Hill-Taylor et al., 2016), while START criteria aim to reduce underprescribing (Cherubini et al., 2012) by identifying PPOs. Meanwhile, Corsonello et al. (2012) reported that the STOPP/START criteria, compared to the Beers criteria, show a greater ability to predict ADRs and prevent potentially inappropriate prescribing. In addition, the STOPP/START criteria seemed to afford a good inter-rater reliability when the evaluations carried out by pharmacists from different sectors were compared (Ryan et al., 2009a). However, for that, it is important to have full access to the clinical information; otherwise, PIMs and PPOs detection can be overestimated and underestimated, respectively (Ryan et al., 2013b).

The STOPP/START criteria have been applied to different target populations of different settings [such as hospital, nursing homes, community-dwelling, primary care, and post-acute care (PAC) and long-term care (LTC)]. For instance, a meta-analysis of 28 studies in elderly patients showed that the prevalence of PIMs and PPOs was high, with the highest values observed in hospitalized patients and nursing homes, compared to community dwelling-individuals for national outpatient databases small community studies (Thomas, 2016). In another meta-analysis, including both PAC and LTC patients, it was demonstrated that the STOPP/START criteria may be effective in improving prescribing quality, clinical, humanistic and economic outcomes (Hill-Taylor et al., 2016). However, while Hill-Taylor et al. (2016) reported less falls, delirium episodes, hospital length-of-stay, care visits, and medication costs, they found no association with improvements in quality of life or mortality. More recent evidence, Thomas et al. (2020) suggests that both PIMs and PPOs were significantly associated with hospital readmission and mortality within 6 months.

In Portugal, there are few examples of investigations using the START/STOPP criteria (Silva et al., 2015; da Costa et al., 2016). However, no one to the best of our knowledge has included the Units for Integrated Continuous Care (*Unidades de Cuidados Continuados*, UCCIs) inserted in the Portuguese National Network for Long-term Integrated Care (*Rede Nacional de Cuidados Continuados Integrados*, RNCCI). Therefore, the present study was carried out to: 1) determine the prevalence of PIMs and PPOs (overall and per individual STOPP and START criteria, respectively); and 2) potential predictors of PIMs and PPOs among demographic and clinical features of elderly patients who received PAC/LTC in UCCIs of the RNCCI.

## Patients and Methods

### Study Design, Setting, and Participants

An observational, retrospective, cross-sectional, multicenter study was performed in 161 patients aged ≥65 years from UCCIs in the central region of Portugal, between June 2015 and April 2016. The UCCIs belong to the category of patient units and provide continuous support to frail people, for rehabilitation in PAC and for people with mental, social, and physical limitations who need LTC. According to each patient needs and goals established, the length of stay usually varied between 30 and 180 consecutive days. All patients are monitored by a multidisciplinary team of various professionals, such as doctors, nurses, pharmacists, physiotherapists, social workers, psychologists, speech therapists, occupational therapists, and nutritionists. To reduce bias associated with the type of hospitalization and healthcare team, the data were collected from eight UCCIs.

The retrospective nature of the study did not affect healthcare provision to patients, and informed consent was not required. Patients’ data were anonymized through the attribution of an alphanumeric code and access restricted to the first author. The subsequent analysis was performed exclusively using the encoded data.

### Data Sources

Data were mainly collected from RNCCI’s platform, which is an online tool implemented in the RNCCI in Portugal. In this platform, all relevant patient information is recorded, namely, discharge summaries, periodic evaluations performed by different professionals (such as physicians, nurses, physiotherapists, psychologists, social workers, and nutritionists), diagnoses, prescribed drugs, medical exams, nutrition status, dependence in activities of daily life, products spent (e.g., ostomy, wound or incontinence products), identification of need for social support and results of medical scales application (e.g., risk of falls, pressure ulcer risk assessment, calculation of the risk of developing type 2 diabetes mellitus in the next 10 years and pain evaluation). In addition, patient clinical history was complemented with other existent documents (e.g., patient diary) whenever possible and necessary.

### Data Collection and Analysis

A detailed analysis was used for each patient by a pharmacist, including demographic and clinical data, namely, all current diagnoses (not only those coded through ICD-9-CM), relevant clinical information reported from the first medical evaluation (before to the actual internment) until discharge and an update on the latest therapeutic list. All pharmaceutical dosage forms including oral, parenteral, topical, ophthalmological, and inhaled medications, taken on a regular basis (excluding SOS medications) were considered. If a fixed-dose combination of drugs was used in the same medication, it was only counted as one. Polypharmacy (intake of ≥5 drugs per day), comorbid diseases, Charlson Comorbidity Index (Charlson et al., 1987) (CCI ≥ 4 and CCI ≥ 6), dependency in activities of daily life (ADL), risk of falls (medium to high), malnutrition/anorexia, obesity, pressure ulcers and history of recent fractures were also considered as geriatric syndromes. Continuous variables were expressed as mean ± standard deviation, median and inter-quartile range (P25; P75), and categorical variables as the number of observations (absolute frequency) and percentages (relative frequency). To identify the determinants of PIMs and PPOs, variables with a significant association with PIMs or PPOs at the univariate level were tested using a multivariate analysis. Logistic regression analysis, with logit link function, was performed using the forward selection method based on the Wald test to find independent predictors associated with PIMs or PPOs. Also, odds ratios (ORs) were adjusted for possible confounding variables, and results were reported only for variables with a *p* < 0.1. The Hosmer-Lemeshow test was performed to assess the goodness of fit, whereas the area under the receiver operating characteristic curve allowed the evaluation of discriminatory power of the model and its sensitivity/specificity. Differences were considered statistically significant when *p* < 0.05 and the confidence interval (CI) was set at 95%. IBM SPSS Statistics version 23 was used to analyse all the data.

## Results

### Characteristics of the Study Population

Table 1 details patients’ demographic characteristics and medical history. From 161 patients 103 were female (64.0%). The average age of patients was 81.6 years and the medical history demonstrated higher provenience from the hospital (50.9%). The median length-of-stay in UCCIs was 93 days and 61 patients returned home (37.9%; Table 1). Of the remaining 100, the highest number either died during the internment (28 patients) or has been transferred to another RNCCI response (28 patients; Table 1). Table 2 demonstrates that inpatients frequently took a median of 9 (P25: 6; P75: 11) drugs per day, totaling a median of 10 (P25: 7; P75: 13) daily oral doses, a CCI median of 6 (P25: 5; P75: 7) and 21 patients were fed by enteral nutrition (13.0%). Regarding geriatric syndromes, 147 patients had polypharmacy, 143 had high levels of dependency and 131 presented risk of falls (91.3, 88.8, and 81.4%, respectively; Table 2). Most common comorbidities were hypertension (68.3%), cerebrovascular disease (34.8%), depression (34.2%), diabetes mellitus (33.5%) and constipation (33.5%; Table 3).

**TABLE 1:** Demographic characteristics and medical history of study population (*N*=161) that received post-acute care and long-term care in Units for Integrated Continuous Care (*Unidades de Cuidados Continuados*, UCCI) inserted in the Portuguese National Network for Long-term Integrated Care (*Rede Nacional de Cuidados Continuados Integrados*, RNCCI).

		STOPP criteria				START criteria			
	Total	PIMs	No PIMs	Not adjusted OR (95%CI)	*p* ^a^	PPOs	No PPOs	Not adjusted OR (95%CI)	*p* ^a^
**Demographic characteristics**									
Age (years)				1.01 (0.95; 1.07)	0.827			1.04 (0.99; 1.10)	0.123
Mean ± SD	81.6 ± 7.4	81.7 ± 7.0	81.3 ± 9.8			82.0 ± 7.4	79.7 ± 7.6		
Median (P25; P75)	82 (76.5; 86.5)	82 (77; 86)	80.5 (74; 88.5)			82 (78; 87)	78.5 (75; 85)		
Gender, *n* (%)									
Male	58 (36.0)	43 (31.4)	15 (62.5)	1		48 (36.6)	10 (33.3)	1	
Female	103 (64.0)	94 (68.6)	9 (37.5)	3.64 (1.48; 8.98)	0.005	83 (63.4)	20 (66.7)	0.87 (0.37; 2.00)	0.734
**Medical history**										
Provenience/Origin, *n* (%)									
Hospital	82 (50.9)	74 (54.0)	8 (33.3)	2.74 (1.09; 6.87)	0.031	65 (49.6)	17 (56.7)	0.79 (0.35; 1.80)	0.575
Residence	70 (43.5)	54 (39.4)	16 (66.7)	1		58 (44.3)	12 (40.0)	1	
Nursing home	5 (3.1)	5 (3.6)	0 (0.0)	—		4 (3.1)	1 (3.3)	0.83 (0.09; 8.07)	0.871
Primary care	2 (1.2)	2 (1.5)	0 (0.0)	—		2 (1.5)	0 (0.0)	—	
Other	2 (1.2)	2 (1.5)	0 (0.0)	—		2 (1.5)	0 (0.0)	—	
Provenience/Origin, *n* (%)									
Hospital	82 (50.9)	74 (54.0)	8 (33.3)	2.35 (0.94; 5.85)	0.067	65 (49.6)	17 (56.7)	0.75 (0.34; 1.68)	0.487
Residence or other	79 (49.1)	63 (46.0)	16 (66.7)	1		66 (50.4)	13 (43.3)	1	
Length of stay				1.00 (1.00; 1.01)	0.182			1.00 (1.00; 1.00)	0.652
Mean ± SD	146.1 ± 190.7	154.8 ± 204.0	96.0 ± 62.3			149.3 ± 183.9	131.8 ± 221.1		
Median (P25; P75)	93 (65; 163.5)	98 (65; 167.5)	90 (68.5; 95)			97 (79; 168)	90 (42.5; 112)		
Discharge to, *n* (%)									
Residence	61 (37.9)	47 (34.3)	14 (58.3)	0.26 (0.05; 1.23)	0.088	44 (33.6)	17 (56.7)	0.52 (0.06; 4.76)	0.561
Death	28 (17.4)	26 (19.0)	2 (8.3)	1.00 (0.13; 7.64)		27 (20.6)	1 (3.3)	5.40 (0.29; 101.28)	0.260
Another RNCCI response	28 (17.4)	26 (19.0)	2 (8.3)	1	1.000	22 (16.8)	6 (20.0)	0.73 (0.07; 7.53)	0.794
Social option/response	20 (12.4)	16 (11.7)	4 (16.7)	0.31 (0.05; 1.88)	0.201	17 (13.0)	3 (10.0)	1.13 (0.10; 13.44)	0.921
Nursing home	17 (10.6)	15 (10.9)	2 (8.3)	0.58 (0.07; 4.53)	0.601	16 (12.2)	1 (3.3)	3.20 (0.17; 61.02)	0.439
Other or not referred	6 (3.7)	6 (4.4)	0 (0.0)	—		5 (3.8)	1 (3.3)	1	
Emergency department	1 (0.6)	1 (0.7)	0 (0.0)	—		0 (0.0)	1 (3.3)	—	

CI, confidence interval; OR, odd ratio; PIMs, potentially inappropriate medications; PPOs, potential prescribing omissions; SD, standard deviation; START, Screening Tool to Alert to Right Treatment; STOPP, Screening Tool of Older People’s Prescriptions.

a Wald test.

**TABLE 2:** Clinical features of study population (*N* = 161) that received post-acute care and long-term care in Units for Integrated Continuous Care (*Unidades de Cuidados Continuados*, UCCI) inserted in the Portuguese National Network for Long-term Integrated Care (*Rede Nacional de Cuidados Continuados Integrados*, RNCCI).

		STOPP criteria				START criteria			
	Total	PIMs	No PIMs	Not adjusted OR (95%CI)	*p* ^a^	PPOs	No PPOs	Not adjusted OR (95%CI)	*p* ^a^
**Clinical features**									
Enteral Nutrition, *n* (%)									
Yes	21 (13.0)	19 (13.9)	2 (8.3)	1.77 (0.39; 8.15)	0.463	19 (14.5)	2 (6.7)	2.38 (0.52; 10.80)	0.263
No	140 (87.0)	118 (86.1)	22 (91.7)	1		112 (85.5)	28 (93.3)	1	
Medication per patient				1.30 (1.11; 1.53)	0.002			1.12 (0.98; 1.27)	0.086
Mean ± SD	8.84 ± 3.32	9.20 ± 3.19	6.79 ± 3.40			9.06 ± 3.38	7.90 ± 2.93		
Median (P25; P75)	9 (6; 11)	9 (7; 11)	7 (4.5; 8)			9 (6; 11)	8 (5; 10)		
Number of doses				1.51 (1.02; 1.30)	0.024			1.04 (0.94; 1.15)	0.407
Mean ± SD	10.20 ± 4.14	10.51 ± 4.03	8.42 ± 4.41			10.33 ± 4.22	9.63 ± 3.79		
Median (P25; P75)	10 (7; 13)	10 (8; 13)	8 (6; 11)			10 (7; 13)	10 (6; 12)		
Comorbid diseases				0.99 (0.68; 1.46)	0.976			2.10 (1.35; 3.29)	0.001
Mean ± SD	1.70 ± 1.14	1.70 ± 1.16	1.71 ± 1.04			1.85 ± 1.15	1.07 ± 0.83		
Median (P25; P75)	2 (1; 2)	2 (1; 2)	2 (1; 2)			2 (1; 3)	1 (0; 2)		
CCI				1.13 (0.87; 1.47)	0.376			1.56 (1.17; 2.06)	0.002
Mean ± SD	5.83 ± 1.71	5.88 ± 1.71	5.54 ± 1.69			6.03 ± 1.66	4.93 ± 1.64		
Median (P25; P75)	6 (5; 7)	6 (5; 7)	5 (4; 6.5)			6 (5; 7)	5 (4; 6)		
Geriatric syndromes, *n* (%)									
Polypharmacy (≥5 drugs/day)									
Yes	147 (91.3)	129 (94.2)	18 (75.0)	5.38 (1.67; 17.28)	0.005	121 (92.4)	26 (86.7)	1.86 (0.54; 6.40)	0.324
No	14 (8.7)	8 (5.8)	6 (25.0)	1		10 (7.6)	4 (13.3)	1	
Comorbid diseases ≥ 2									
Yes	86 (53.4)	73 (53.3)	13 (54.2)	0.97 (0.40; 2.30)	0.936	77 (58.8)	9 (30.0)	3.33 (1.42; 7.82)	0.006
No	75 (46.6)	64 (46.7)	11 (45.8)	1		54 (41.2)	21 (70.0)	1	
CCI ≥ 4									
Yes	149 (92.5)	127 (92.7)	22 (91.7)	1.16 (0.24; 5.63)	0.859	124 (94.7)	25 (83.3)	3.54 (1.04; 12.07)	0.043
No	12 (7.5)	10 (7.3)	2 (8.3)	1		7 (5.3)	5 (16.7)	1	
CCI ≥ 6									
Yes	85 (52.8)	74 (54.0)	11 (45.8)	1.39 (0.58; 3.32)	0.460	76 (58.0)	9 (30.0)	3.22 (1.37; 7.58)	0.007
No	76 (47.2)	63 (46.0)	13 (54.2)	1		55 (42.0)	21 (70.0)	1	
Dependency in ADL									
Yes	143 (88.8)	120 (87.6)	23 (95.8)	0.31 (0.04; 2.42)	0.262	120 (91.6)	23 (76.7)	3.32 (1.17; 9.46)	0.025
No	18 (11.2)	17 (12.4)	1 (4.2)	1		11 (8.4)	7 (23.3)	1	
Fall Risk (medium or high)									
Yes	131 (81.4)	113 (82.59	18 (75.0)	1.57 (0.56; 4.37)	0.388	109 (83.2)	122(73.3)	1.80 (0.71; 4.57)	0.215
No	30 (18.6)	24 (17.5)	6 (25.0)	1		22 (16.8)	8 (26.7)	1	
Malnutrition/anorexia									
Yes	7 (4.3)	5 (3.6)	2 (8.3)	0.42 (0.08; 2.28)	0.313	5 (3.8)	2 (6.7)	0.56 (0.10; 3.01)	0.495
No	154 (95.7)	132 (96.4)	22 (91.7)	1		126 (96.2)	28 (93.3)	1	
Obesity									
Yes	22 (13.7)	19 (13.9)	3 (12.5)	1.13 (0.31; 4.15)	0.857	17 (13.0)	5 (16.7)	0.75 (0.25; 2.21)	0.597
No	139 (86.3)	118 (86.1)	21 (87.5)	1		114 (87.0)	25 (83.3)	1	
Pressure ulcers at discharge									
Yes	27 (16.8)	25 (18.2)	2 (8.3)	2.46 (0.54; 11.13)	0.244	24 (18.3)	3 (10.0)	2.02 (0.57; 7.21)	0.279
No	134 (83.2)	112 (81.8)	22 (91.7)	1		107 (81.7)	27 (90.0)	1	
History of recent fractures									
Yes	46 (28.6)	39 (28.5)	7 (29.2)	0.97 (0.37; 2.51)	0.944	44 (33.6)	2 (6.7)	7.07 (1.61; 31.09)	0.010
No	115 (71.4)	98 (71.5)	17 (70.8)	1		87 (66.4)	28 (93.3)	1	

ADL, dependency in activities of daily life; CCI, Charlson Comorbidity Index; CI, confidence interval; PIMs, potentially inappropriate medications; PPOs, potential prescribing omissions; OR, odd ratio; SD, standard deviation; START, Screening Tool to Alert to Right Treatment; STOPP, Screening Tool of Older People’s Prescriptions.

a Wald test.

**TABLE 3:** Most common/significant comorbidities of study population (*N* = 161) that received post-acute care and long-term care in Units for Integrated Continuous Care (*Unidades de Cuidados Continuados*, UCCI) inserted in the Portuguese National Network for Long-term Integrated Care (*Rede Nacional de Cuidados Continuados Integrados*, RNCCI).

Most common/significant comorbidities, *n* (%)		STOPP criteria				START criteria			
	Total	PIMs	No PIMs	Not adjusted OR (95%CI)	*p* ^a^	PPOs	No PPOs	Not adjusted OR (95%CI)	*p* ^a^
Hypertension									
Yes	110 (68.3)	96 (70.1)	14 (58.3)	1.67 (0.69; 4.07)	0.257	92 (70.2)	18 (60.0)	1.57 (0.69; 3.57)	0.280
No	51 (31.7)	41 (29.9)	10 (41.7)	1		39 (29.8)	12 (40.0)	1	
Cerebrovascular disease									
Yes	56 (34.8)	42 (30.7)	14 (58.3)	0.32 (0.13; 0.77)	0.011	47 (35.9)	9 (30.0)	1.31 (0.55; 3.08)	0.543
No	105 (65.2)	95 (69.3)	10 (41.7)	1		84 (64.1)	21 (70.0)	1	
Depression									
Yes	55 (34.2)	52 (38.0)	3 (12.5)	4.28 (1.22; 15.07)	0.023	45 (34.4)	10 (33.3)	1.05 (0.45; 2.43)	0.916
No	106 (65.8)	85 (62.0)	21 (87.5)	1		86 (65.6)	20 (66.7)	1	
Diabetes mellitus									
Yes	54 (33.5)	45 (32.8)	9 (37.5)	0.82 (0.33; 2.01)	0.656	46 (35.1)	8 (26.7)	1.49 (0.61; 3.61)	0.379
No	107 (66.5)	92 (67.2)	15 (62.5)	1		85 (64.9)	22 (73.3)	1	
Constipation									
Yes	54 (33.5)	51 (37.2)	3 (12.5)	4.15 (1.18; 14.61)	0.027	47 (35.9)	7 (23.3)	1.84 (0.73; 4.61)	0.194
No	107 (66.5)	86 (62.8)	21 (87.5)	1		84 (64.1)	23 (76.7)	1	
Dementia									
Yes	47 (29.2)	43 (31.4)	4 (16.7)	2.29 (0.74; 7.10)	0.152	43 (32.8)	4 (13.3)	3.18 (1.04; 9.68)	0.042
No	114 (70.8)	94 (68.6)	20 (83.3)	1		88 (67.2)	26 (86.7)	1	
Urinary incontinence									
Yes	45 (28.0)	42 (30.7)	3 (12.5)	3.10 (0.88; 10.94)	0.080	41 (31.3)	4 (13.3)	2.96 (0.97; 9.04)	0.056
No	116 (72.0)	95 (69.3)	21 (87.5)	1		90 (68.7)	26 (86.7)	1	
Rheumatic Disease									
Yes	38 (23.6)	31 (22.6)	7 (29.2)	0.71 (0.27; 1.87)	0.488	29 (22.1)	9 (30.0)	0.66 (0.27; 1.60)	0.362
No	123 (76.4)	106 (77.4)	17 (70.8)	1		102 (77.9)	21 (70.0)	1	
Congestive heart failure									
Yes	36 (22.4)	32 (23.4)	4 (16.7)	1.52 (0.49; 4.78)	0.471	34 (26.0)	2 (6.7)	4.91 (1.11; 21.70)	0.036
No	125 (77.6)	105 (76.6)	20 (83.3)	1		97 (74.0)	28 (93.3)	1	
Arrhythmia									
Yes	29 (18.0)	26 (19.0)	3 (12.5)	1.64 (0.46; 5.91)	0.450	29 (22.1)	0 (0.0)	—	—
No	132 (82.0)	111 (81.0)	21 (87.5)	1		102 (77.9)	30 (100.0)		
Benign prostatic hypertrophy									
Yes	28 (48.3)	21 (48.8)	7 (46.7)	1.09 (0.34; 3.54)	0.885	27 (56.3)	1 (10.0)	11.57 (1.36; 98.67)	0.025
No	30 (51.7)	22 (51.2)	8 (53.3)	1		21 (43.8)	9 (90.0)	1	
Renal disease									
Yes	23 (14.3)	21 (15.3)	2 (8.3)	1.99 (0.44; 9.11)	0.375	20 (15.3)	3 (10.0)	1.62 (0.45; 5.86)	0.461
No	138 (85.7)	116 (84.7)	22 (91.7)	1		111 (84.7)	27 (90.0)	1	
Chronic pulmonary obstructive disease									
Yes	20 (12.4)	15 (10.9)	5 (20.8)	0.47 (0.15; 1.43)	0.184	19 (14.5)	1 (3.3)	4.92 (0.63; 38.29)	0.128
No	141 (87.6)	122 (89.1)	19 (79.2)	1		112 (85.5)	29 (96.7)	1	
Non-metastatic solid tumor									
Yes	20 (12.4)	19 (13.9)	1 (4.2)	3.70 (0.47; 29.05)	0.213	16 (12.2)	4 (13.3)	0.90 (0.28; 2.93)	0.867
No	141 (87.6)	118 (86.1)	23 (95.8)	1		115 (87.8)	26 (86.7)	1	
Hemiplegia									
Yes	15 (9.3)	12 (8.8)	3 (12.5)	0.67 (0.18; 2.58)	0.563	13 (9.9)	2 (6.7)	1.54 (0.33; 7.23)	0.582
No	146 (90.7)	125 (91.2)	21 (87.5)	1		118 (90.1)	28 (93.3)	1	
Parkinson’s disease									
Yes	6 (3.7)	3 (2.2)	3 (12.5)	0.16 (0.03; 0.83)	0.029	3 (2.3)	3 (10.0)	0.21 (0.04; 1.10)	0.065
No	155 (96.3)	134 (97.8)	21 (87.5)	1		128 (97.7)	27 (90.0)	1	
Metastatic solid tumor									
Yes	5 (3.1)	5 (3.6)	0 (0.0)	—	—	4 (3.1)	1 (3.3)	0.91 (0.10; 8.48)	0.936
No	156 (96.9)	132 (96.4)	24 (100.0)			127 (96.9)	29 (96.7)	1	
Angina									
Yes	4 (2.5)	3 (2.2)	1 (4.2)	0.52 (0.05; 5.17)	0.573	3 (2.3)	1 (3.3)	0.68 (0.07; 6.77)	0.742
No	157 (97.5)	134 (97.8)	23 (95.8)	1		128 (97.7)	29 (96.7)	1	
Osteoporosis									
Yes	3 (1.9)	3 (2.2)	0 (0.0)	—	—	2 (1.5)	1 (3.3)	0.45 (0.04; 5.13)	0.520
No	158 (98.1)	134 (97.8)	24 (100.0)			129 (98.5)	29 (96.7)	1	158 (98.1)
Glaucoma									
Yes	3 (1.9)	3 (2.2)	0 (0.0)	—	—	2 (1.5)	1 (3.3)	0.45 (0.04; 5.13)	0.520
No	158 (98.1)	134 (97.8)	24 (100.0)			129 (98.5)	29 (96.7)	1	

CI, confidence interval; PIMs, potentially inappropriate medications; PPOs, potential prescribing omissions; OR, odd ratio; SD, standard deviation; START, Screening Tool to Alert to Right Treatment; STOPP, Screening Tool of Older People’s Prescriptions.

a Wald test.

### Potentially Inappropriate Medications

According to STOPP criteria, patients had a median of 3 [1; 4] PIMs (range 0–10), with 85.1% of them presenting at least one and about a fifth had five or more PIMs in their list of prescriptions (Table 4). Sections with higher frequency of PIMs were found in “Central Nervous System and psychotropic drugs” (66.5%) and “drugs that predictably increase the risk of falls in older people” (65.8%). Among “Central Nervous System and psychotropic drugs” section, the most common PIMs in patients were benzodiazepines for ≥4 weeks (D5; 51.6%; Table 5), tricyclic antidepressants with dementia, narrow angle glaucoma, cardiac conduction abnormalities, prostatism, or prior history of urinary retention (D1; 15.5%; Table 5), and anticholinergics/antimuscarinics in patients with delirium or dementia (D7; 13.7%; Table 5). Among “drugs that predictably increase the risk of falls in older people” were benzodiazepines (K1; 54.0% Table 5) and neuroleptics (K2; 24.8%; Table 5).

**TABLE 4:** Number of potentially inappropriate medications (PIMs) and potential prescribing omissions (PPOs), according to Screening Tool of Older People’s Prescriptions (STOPP) and Screening Tool to Alert to Right Treatment (START) criteria, respectively (*N* = 161).

Number of PIMs/PPOs	STOPP criteria (*n*, %)	START criteria (*n*, %)
0	24 (14.9)	30 (18.6)
1	23 (14.3)	36 (22.4)
2	32 (19.9)	47 (29.2)
3	33 (20.5)	28 (17.4)
4	16 (9.9)	13 (8.1)
5	16 (20.5)	6 (3.7)
6	10 (6.2)	1 (0.6)
≥7	7 (4.3)	0 (0.0)
Total	137 (85.1)	131 (81.4)
Mean ± SD	2.8 ± 2.1	1.9 ± 1.4
Median (P25; P75)	3 [1; 4]	2 [1; 3]

SD, standard deviation.

**TABLE 5:** Frequency of potentially inappropriate medications (PIMs) and potential prescribing omissions (PPOs) according to Screening Tool of Older People’s Prescriptions (STOPP) and Screening Tool to Alert to Right Treatment (START) criteria respectively (*N* = 161).

			*n* (%)
**STOPP criteria**	A	Indication of medication	27 (16.8)
	A1	Any drug prescribed without an evidence-based clinical indication.	15 (9.3)
	A3	Any duplicate drug class prescription.	15 (9.3)
	B	Cardiovascular System	28 (17.4)
	B9	Loop diuretic for treatment of hypertension with concurrent urinary incontinence.	10 (6.2)
	B12	Aldosterone antagonists with concurrent potassium-conserving drugs without monitoring of serum potassium.	7 (4.3)
	C	Antiplatelet/Anticoagulant Drugs	9 (5.6)
	C7	Ticlopidine in any circumstances.	5 (3.1)
	D	Central Nervous System and Psychotropic Drugs	107 (66.5)
	D1	Tricyclic antidepressants with dementia, narrow angle glaucoma, cardiac conduction abnormalities, prostatism, or prior history of urinary retention.	25 (15.5)
	D2	Initiation of tricyclic antidepressants as first-line antidepressant treatment.	11 (6.8)
	D5	Benzodiazepines for ≥ 4 weeks.	83 (51.6)
	D7	Anticholinergics/antimuscarinics in patients with delirium or dementia.	22 (13.7)
	D9	Neuroleptic antipsychotic in patients with behavioural and psychological symptoms of dementia unless symptoms are severe and other treatments have failed.	8 (5.0)
	D11	Acetylcholinesterase inhibitors with a known history of persistent bradycardia heart block or recurrent unexplained syncope or concurrent treatment with drugs that reduce heart rate.	6 (3.7)
	D14	First-generation antihistamines.	8 (5.0)
	E	Renal System	0 (0.0)^a^
	F	Gastrointestinal System	22 (17.7)
	F2	Proton-pump inhibitors for uncomplicated peptic ulcer disease or erosive peptic oesophagitis at full therapeutic dosage for > 8 weeks.	10 (6.2)
	F3	Drugs likely to cause constipation in patients with chronic constipation where non-constipating alternatives are appropriate.	12 (7.5)
	G	Respiratory System	14 (8.7)
	G5	Benzodiazepines with acute or chronic respiratory failure.	13 (8.1)
	H	Musculoskeletal System	1 (0.2)
	I	Urogenital System	1 (0.2)
	J	Endocrine System	0 (0.0)^a^
	K	Drugs that predictably increase the risk of falls in older people	106 (65.8)
	K1	Benzodiazepines	87 (54.0)
	K2	Neuroleptic drugs	40 (24.8)
	K4	Hypnotic Z-drugs	9 (5.6)
	L	Analgesic Drugs	18 (11.2)
	L2	Use of regular (as distinct from *pro re nata*) opioids without concomitant laxative.	12
	L3	Long-acting opioids without short-acting opioids for break-through pain.	8 (5.0)
	N	Antimuscarinic/Anticholinergic Drug Burden	8 (5.0)
**START criteria**	A	Cardiovascular System	64 (39.8)
	A1	Vitamin K antagonists or direct thrombin inhibitors or factor Xa inhibitors in the presence of chronic atrial fibrillation.	5 (3.1)
	A3	Antiplatelet therapy (aspirin or clopidogrel or prasugrel or ticagrelor) with a documented history of coronary, cerebral or peripheral vascular disease.	22 (13.7)
	A5	Statin therapy with a documented history of coronary, cerebral or peripheral vascular disease, unless the patient’s status is end-of-life or age is >85 years.	15 (9.3)
	A6	Angiotensin Converting Enzyme inhibitor with systolic heart failure and/or documented coronary artery disease.	28 (17.4)
	A8	Appropriate beta-blocker (bisoprolol, nebivolol, metoprolol or carvedilol) with stable systolic heart failure.	18 (11.2)
	B	Respiratory System	12 (7.5)
	B1	Regular inhaled 2 agonist or antimuscarinic bronchodilator (e.g., ipratropium, tiotropium) for mild to moderate asthma or chronic obstructive pulmonary disease.	10 (6.1)
	C	Central Nervous System and Eyes	19 (11.8)
	C2	Non-tricyclic antidepressant drug in the presence of persistent major depressive symptoms.	12 (7.5)
	D	Gastrointestinal System	0 (0.0)^a^
	E	Musculoskeletal System	89 (55.3)
	E3	Vitamin D and calcium supplement in patients with known osteoporosis and/or previous fragility fracture(s) and/or Bone Mineral Density T-scores.	44 (27.3)
	E5	Vitamin D supplement in older people who are housebound or experiencing falls or with osteopenia.	74 (46.0)
	F	Endocrine System	6 (3.7)
	F1	Angiotensin Converting Enzyme inhibitor or Angiotensin Receptor Blocker (if intolerant of Angiotensin Converting Enzyme inhibitor) in diabetes with evidence of renal disease, i.e., dipstick proteinuria or microalbuminuria (>30 mg/24 hours) with or without serum biochemical renal impairment.	6 (3.7)
	G	Urogenital System	22 (13.7)
	G1	Alpha-1 receptor blocker with symptomatic prostatism, where prostatectomy is not considered necessary.	12 (7.5)
	G2	5-alpha reductase inhibitor with symptomatic prostatism, where prostatectomy is not considered necessary.	18 (11.2)
	H	Analgesics	12 (7.5)
	H2	Laxatives in patients receiving opioids regularly.	12 (7.5)
	I	Vaccines	0 (0.0)^a^

a Not applicable.

In the multivariate analysis (Table 6), PIMs were found to be significantly associated with gender (F/M) (OR = 4.04, 95%CI: 1.27; 12.84), hospital provenience (OR = 3.43, 95%CI: 1.10; 10.69), number of medications (OR = 1.32, 95%CI: 1.09; 1.60), cerebrovascular disease (OR = 0.29, 95%CI: 0.10; 0.89) and Parkinson’s disease (OR = 0.06, 95%CI: 0.00; 0.84).

**TABLE 6:** Predictors of potentially inappropriate medications (PIMs), according to Screening Tool of Older People’s Prescriptions (STOPP) criteria, in the study population (*N* = 161).

	Total	PIM	No PIM	Adjusted OR (95% CI)	*p* ^a^
Gender, *n* (%)					
Male	58 (36.0)	43 (31.4)	15 (62.5)	1	
Female	103 (64.0)	94 (68.6)	9 (37.5)	4.04 (1.27; 12.84)	0.018
Provenience/Origin, *n* (%)					
Hospital	91 (56.5)	83 (60.6)	8 (33.3)	3.43 (1.10; 10.69)	0.034
Residence or other	70 (43.5)	54 (39.4)	16 (66.7)	1	
Medication per patient				1.32 (1.09; 1.60)	0.005
Mean ± SD	8.84 ± 3.32	9.20 ± 3.19	6.79 ± 3.40		
Median (P25; P75)	9 (6; 11)	9 (7; 11)	7 (4.5; 8)		
History of recent fractures					
Yes	46 (28.6)	39 (28.5)	7 (29.2)	0.31 (0.09; 1.06)	0.062
No	115 (71.4)	98 (71.5)	17 (70.8)	1	
Cerebrovascular disease					
Yes	56 (34.8)	42 (30.7)	14 (58.3)	0.29 (0.10; 0.89)	0.030
No	105 (65.2)	95 (69.3)	10 (41.7)	1	
Depression					
Yes	55 (34.2)	52 (38.0)	3 (12.5)	4.02 (0.88; 18.42)	0.073
No	106 (65.8)	85 (62.0)	21 (87.5)	1	
Dementia					
Yes	47 (29.2)	43 (31.4)	4 (16.7)	4.62 (0.98; 21.85)	0.054
No	114 (70.8)	94 (68.6)	20 (83.3)	1	
Parkinson’s disease					
Yes	6 (3.7)	3 (2.2)	3 (12.5)	0.06 (0.00; 0.84)	0.037
No	155 (96.3)	134 (97.8)	21 (87.5)	1	

CI, confidence interval; OR, odd ratio; SD, standard deviation.

a Wald test; OR’s adjust with all the variables of Tables 1–3 without null frequencies, but we only show the results for the variables that *p* < 0.1; Omnibus test: *p* < 0.001; Hosmer and Lemeshow test: *p* = 0.291; area under the receiver operating characteristic curve = 0.866 [95% CI: (0.801; 0.931), *p* < 0.001]; Sensitivity = 79.6% and Specificity = 87.5% are simultaneously maximized for the cutoff probability 0.8109.

### Potential Prescribing Omissions

According to START criteria, patients had a median of 2 [1; 3] PPOs (range 0–6), with 81.4% of them having at least one PPO and more than half of patients had one or two PPOs (Table 4). Most associated systems with PPOs were “Musculoskeletal System” (55.3%) and “Cardiovascular System” (39.8%). In the “Musculoskeletal System”, the highest frequency of PPOs was associated with “vitamin D supplementation in elderly people who are housebound or experiencing falls or with osteopenia” (E5; 46%; Table 5) followed by “vitamin D and calcium supplement in patients with known osteoporosis and/or previous fragility fracture(s) and/or Bone Mineral Density T-scores more than −2.5 in multiple sites” (E3; 27.3%; Table 5). Among “Cardiovascular System” the highest frequency of PPOs was associated with “angiotensin-converting enzyme inhibitor with systolic heart failure and/or documented coronary artery disease” (A6; 17.4%; Table 5), and “antiplatelet therapy (aspirin, clopidogrel, prasugrel or ticagrelor, with a documented history of coronary, cerebral or peripheral vascular disease)” (A3; 13.7%; Table 5).

In the multivariate analysis (Table 7), PPOs were found to be independently associated with the number of CCI (OR = 2.14, 95%CI: 1.46; 3.14), history of recent fractures (OR = 13.90, 95%CI: 2.83; 68.36), Parkinson’s disease (OR = 0.08, 95%CI: 0.01; 0.61) and metastatic solid tumor (OR = 0.03, 95%CI: 0.00; 0.59).

**TABLE 7:** Predictors of potential prescribing omissions (PPOs), according to Screening Tool to Alert to Right Treatment (START) criteria, in the study population (*N* = 161).

	Total	PPOs	No PPOs	Adjusted OR (95% CI)	*p* ^a^
Gender, *n* (%)					
Male	58 (36.0)	48 (36.6)	10 (33.3)	1	
Female	103 (64.0)	83 (63.4)	20 (66.7)	0.38 (0.14; 1.05)	0.063
CCI				2.14 (1.46; 3.14)	<0.001
Mean ± SD	5.83 ± 1.71	6.03 ± 1.66	4.93 ± 1.64		
Median (P25; P75)	6 (5; 7)	6 (5; 7)	5 (4; 6)		
History of recent fractures					
Yes	45 (28.0)	41 (31.3)	4 (13.3)	13.90 (2.83; 68.36)	0.001
No	116 (72.0)	90 (68.7)	26 (86.7)	1	
Non-metastatic solid tumor					
Yes	20 (12.4)	16 (12.2)	4 (13.3)	0.29 (0.07; 1.26)	0.099
No	141 (87.6)	115 (87.8)	26 (86.7)	1	
Parkinson’s disease					
Yes	6 (3.7)	3 (2.3)	3 (10.0)	0.08 (0.01; 0.61)	0.015
No	155 (96.3)	128 (97.7)	27 (90.0)	1	
Metastatic solid tumor					
Yes	5 (3.1)	4 (3.1)	1 (3.3)	0.03 (0.00; 0.59)	0.021
No	156 (96.9)	127 (96.9)	29 (96.7)	1	

CCI, Charlson Comorbidity Index; CI, confidence interval, OR, odd ratio; SD, standard deviation.

a Wald test; OR’s adjust with all the variables of Tables 1–3 without null frequencies, but we only show the results for the variables that *p* < 0.1; Omnibus test: *p* < 0.001; Hosmer and Lemeshow test: *p* = 0.744; area under the receiver operating characteristic curve = 0.826 [95% CI: (0.747; 0.905), *p* < 0.001]; Sensitivity = 77.9% and Specificity = 76.7% are simultaneously maximized for the cutoff probability 0.7631.

## Discussion

### Main Findings

The prevalence among inpatients was similar for PIMs (85.1%) and PPOs (81.4%), considering the application of the STOPP and START criteria, respectively. The most involved drugs in PIMs were from the central nervous system group, while PPOs were associated with drugs from the musculoskeletal and cardiovascular system groups. The most common overuses were associated with benzodiazepines as a predictable increase in the risk of falls and when used for longer than 4 weeks. Omissions were more frequently related to the lack of vitamin D supplements, calcium-vitamin D supplements, angiotensin-converting enzyme inhibitors, and antiplatelet agents. Female gender, hospital provenience, and a higher number of prescription drugs were found to be associated with a higher risk for PIMs. In contrast, patients with cerebrovascular disease and Parkinson’s disease had the lowest risk of PIMs. On the other hand, patients with a higher value of CCI and with recent fractures had a higher risk for PPOs, while Parkinson’s disease and metastatic solid tumors were shown to be protective diagnoses for PPOs.

Considering the main findings obtained in our study, it should be highlighted that the number of PIMs per patient (2.8) is lower, but the number of PPOs per patient is higher (1.9), than the reported in a recent study focused on patients admitted to acute care hospitals (3.55 and 0.72, respectively) (Thomas and Nguyen, 2020). On the other hand, the prevalence of PIMs detected in our study (85.1%) is higher than that reported in the literature, in which it ranges from patients 35–77% in patients ≥65 years old (Gallagher and O'Mahony, 2008; Lang et al., 2010; Gallagher P. et al., 2011; Dalleur et al., 2012; Liu et al., 2012; Wahab et al., 2012; Frankenthal et al., 2013; Tosato et al., 2014; San-Jose et al., 2015; Thomas and Thomas, 2019). A higher prevalence of PPOs was also found in our study (81.4%), since the reported values in literature ranged from 34 to 65% (Barry et al., 2007; Lang et al., 2010; Gallagher P. et al., 2011; Dalleur et al., 2012; Liu et al., 2012; Frankenthal et al., 2013; San-Jose et al., 2015). However, PIM rates vary according to each setting: 15–46% in community-dwelling (Galvin et al., 2014; Hedna et al., 2015; Thomas and Thomas, 2019), 21–38% in primary care (Ryan et al., 2009b; Cahir et al., 2010; Bradley et al., 2012; Bradley et al., 2014; Castillo-Paramo et al., 2014; Vezmar Kovacevic et al., 2014), and 48–79% in nursing homes (Garcia-Gollarte et al., 2012; Ubeda et al., 2012; Ryan et al., 2013a); and the same pattern was reported for PPO rates: 30% in community-dwelling (Galvin et al., 2014), 23–51% in primary care (Ryan et al., 2009b; Castillo-Paramo et al., 2014; Vezmar Kovacevic et al., 2014), and 42–74% in nursing homes (Garcia-Gollarte et al., 2012; Ubeda et al., 2012; Ryan et al., 2013a)). Regarding national data, the application of the STOPP/START criteria is scarce. However, Borges et al. (2012) have already identified PPOs in 68% of 91 elderly patients admitted to a stroke unit, Moraes et al. (2013) reported a prevalence of PIMs and PPOs of 74 and 29%, respectively, in 100 patients admitted to a hospital and da Costa et al. (2016) reported PIMs and PPOs of 75 and 43%, respectively, in 161 elderly patients in nursing homes.

Although the prevalence of PIMs and PPOs is generally higher than that reported in the literature, some underlying aspects of existing studies could make this comparison difficult. For instance, Gallagher P. et al. (2011) found a total PIMs prevalence of 51.3% and a global PPOs prevalence of 59.4% considering six European hospitals, but individually different results were observed, for instance a PIMs prevalence of 77.3% in Geneva and a PPOs prevalence of 72.7% in Perugia. In addition, some studies only applied a subset of the STOPP/START criteria (Wahab et al., 2012; Bradley et al., 2014; Galvin et al., 2014), which can result in lower prevalence (Bradley et al., 2014) and misleading direct comparisons. Thus, pulling out the three most frequent PIMs (D5, K1 and K2) and PPOs (A6, E3 and E5) the results would be substantially lower (69 and 60%, respectively). Moreover, of the 81 STOPP criteria, the three most prevalent (D5, K1 and K2) accounted for almost half (47%) of the total of PIMs detected (445). The same happened for the START criteria, with the three most prevalent (A6, E3 and E5) of the 34 criteria accounting for 47% of the total PPOs detected (302). Finally, there are also factors considered by several studies as predictors for PIMs and PPOs that assumed high prevalence in the study population and may contribute to the PIM and PPO rates, such as the number of daily medications [median of 9 (6; 11)], which are higher than those in other studies (Gallagher and O'Mahony, 2008; Ryan et al., 2009b; Lang et al., 2010; Liu et al., 2012; Ubeda et al., 2012; Moraes et al., 2013; Ryan et al., 2013a; Castillo-Paramo et al., 2014); the Charlson Comorbidity Index (CCI) [median of 6 (5; 7)] is also higher than in published data (Gallagher P. et al., 2011; Frankenthal et al., 2013; Castillo-Paramo et al., 2014).

Concerning to most common PIMs, the results are consistent with literature that has reported benzodiazepines (Cahir et al., 2010; Bradley et al., 2012; Dalleur et al., 2012; Garcia-Gollarte et al., 2012; Liu et al., 2012; Ubeda et al., 2012; Wahab et al., 2012; Vezmar Kovacevic et al., 2014; San-Jose et al., 2015), neuroleptics (Garcia-Gollarte et al., 2012; Liu et al., 2012; Bradley et al., 2014), tricyclic antidepressants, anticholinergic/antimuscarinic drugs (Garcia-Gollarte et al., 2012), loop diuretics and proton-pump inhibitors (Cahir et al., 2010; Bradley et al., 2012; Garcia-Gollarte et al., 2012; Wahab et al., 2012; Bradley et al., 2014) as the drug classes mainly involved. The analysis of drugs commonly associated with PPOs is also similar to several other studies that have reported vitamin D (Pyszka et al., 2010), vitamin D and calcium (Barry et al., 2007; Lang et al., 2010; Dalleur et al., 2012; Garcia-Gollarte et al., 2012; Ubeda et al., 2012; San-Jose et al., 2015), angiotensin-converting enzyme inhibitors (Pyszka et al., 2010; Liu et al., 2012), antiplatelet therapy (Barry et al., 2007; Liu et al., 2012), beta-blockers, 5-alpha reductase, statins (Barry et al., 2007; Pyszka et al., 2010; Dalleur et al., 2012; Garcia-Gollarte et al., 2012; Liu et al., 2012), laxatives, alpha-1 receptor blockers and non-tricyclic antidepressants (Lang et al., 2010) as more frequent PPOs.

Relatively to the predictors of PIMs, in our study and also in the literature, female gender has been frequently associated with PIMs (Nyborg et al., 2012; Martins et al., 2015; Barry et al., 2016). Polypharmacy is also commonly identified as a PIM predictor, either as an intake of ≥4 drugs (Bradley et al., 2014; Vezmar Kovacevic et al., 2014), ≥5 drugs (Bradley et al., 2012; Galvin et al., 2014), ≥10 drugs (Gallagher P. et al., 2011; San-Jose et al., 2015) or an increased number of medications (Lang et al., 2010; Wahab et al., 2012; Ryan et al., 2013a; Frankenthal et al., 2013; Castillo-Paramo et al., 2014). The hospital provenience of the patients was not directly tested, but living in an institutional setting was recognized as a predictor of PIMs (Lang et al., 2010), as well as a longer stay at the nursing home (Chen et al., 2012). Among comorbidities, depression is mentioned in the literature (Azermai et al., 2011) but only had a significant association with OR non-adjusted; cerebrovascular disease seemed to be a protective factor, which may be related to a higher supervision or more frequent revision of the therapeutic list of these patients (Zhang et al., 2009); and Parkinson’s disease was also considered to be a protective factor, but no valid reason was found.

Regarding PPOs, they were associated with high values of CCI, in accordance with the literature because the most frequently mentioned factors are comorbidity (CCI) (Frankenthal et al., 2013; Castillo-Paramo et al., 2014), the CCI values higher or equal to 2 (Gallagher P. et al., 2011; Lang et al., 2012), and also multimorbidity (Lang et al., 2010; San-Jose et al., 2015). Fractures have also been cited as predictors (Dalleur et al., 2012) but diagnoses of Parkinson’s disease and metastatic solid tumors are the main findings as protective determinants of PPOs.

Although no other predictors were found, it has been further reported in the literature a history of falls and previous hospitalizations for PIMs (Lang et al., 2010; Frankenthal et al., 2013), and being aged ≥75 years (Vezmar Kovacevic et al., 2014) or ≥85 years (Gallagher P. et al., 2011) for PPOs.

### Strengths and Limitations

The utilization of a common online electronic health platform is an advantage, which permits access to diverse data from all healthcare units included in the sample, such as discharge summaries and several evaluations of the patient from different professionals that allow identification of major clinical data (such as diagnosis, medical history, list of drugs, periodic evaluations, dependency status) and scales for pain evaluation and risk of falls, which help to analyze criteria such as analgesic drugs and the need for calcium-vitamin D supplements. However, the inclusion of eight different healthcare units implies the analysis of eight different multidisciplinary teams that detail information in different ways and fields and, therefore, certain data were sometimes incomplete or even nonexistent; in some cases, it was possible to fill it through internal medical records, other online tools or by information from other settings where the patient was evaluated. Thus, improved access to patients’ information could reduce the time to collect the necessary data to apply medication review criteria and contribute to a larger sample that could allow obtaining better confidence intervals and would be more representative of the Portuguese population and elderly patients receiving PAC/LTC.

Studies have already shown that STOPP/START criteria have good inter-rater reliability between multiple physicians practicing in different centers of Europe (Ryan et al., 2009a; Gallagher et al., 2009); however, it can be difficult to obtain an unequivocal and unquestionable application of certain criteria. Limited length-of-stay, lack of specific medical information or even the interpretation of some criteria led to several limitations, comments, and suggestions regarding the application of STOPP/START criteria discussed along with the study. For instance, it is difficult to understand whether the behavioral and psychological characteristics of dementia are severe enough to justify the use of neuroleptic antipsychotics or to have 100% certainty that a sleep disorder is due to psychosis or dementia. Furthermore, it may not be easy to find alternative drugs for chronic pain treatment in cases of opioid-induced constipation or to ensure that there is no relevance of having a proton-pump inhibitor prescribed in a polymedicated patient with a history of peptic ulcer.

### Implications for Research and/or Practice

Overall, STOPP/START criteria are easy, practical, and fast to apply. Considering the results obtained herein, STOPP/START criteria proved to be a suitable tool for use in PAC/LTC settings, as it has also been internationally demonstrated in other clinical settings. Ryan et al. (2013b) concluded that there is an overestimation of PIMs and an underestimation of PPOs if both criteria are used in the absence of sufficient clinical information. Therefore, the availability of detailed clinical data chronologically organized is essential, as well as drug lists that have complete information (dose, dosage, dosage forms, and administration route and frequency). Besides, the codification of diagnosis and medications by international classifications used worldwide (ATC and ICD-9-CM) would guarantee the universality of the results and would improve comparisons regardless of nationality.

### Future Perspectives

In Portugal, it is imperative to perform studies at larger scales and across all levels of healthcare response, not only to evaluate the national prevalence of PIMs and PPOs but, more importantly, to understand if the trend of existing studies remains high compared to international literature. For these could be important to incentive the pharmacists to introduce the information related to the medication in the online platform that is used by all UCCIs at a national level. In addition, alerts could be programmed to identify PIMs and PPOs, similarly to what happens with the software SENATOR®.

More intensive pharmaceutical interventions can substantially reduce the frequency of PIMs and PPOs, which were already exposed in interventional studies focusing on different healthcare settings (Gallagher PF. et al., 2011; Lang et al., 2012; Dalleur et al., 2014; Frankenthal et al., 2014; Garcia-Gollarte et al., 2014). Lang et al. (Lang et al., 2012) obtained a decrease from 77 to 19% for PIMs and 65–11% for PPOs. Moreover, Garcia-Gollarte et al. (2014) achieved a PIM reduction from 67 to 44% in the intervention group.

It is also crucial to evaluate the compatibility of the application of STOPP and START criteria with the available data from electronic settings [as recently it was made in the US for nursing homes (Khodyakov et al., 2016)], and to improve databases, by modifying or adding relevant information indispensable to apply these criteria. Furthermore, it would be also essential to create a Portuguese version of STOPP/START criteria, as already done in other countries (Delgado Silveira et al., 2015; Lang et al., 2015), and to adapt it to the national market, which would involve modifications in some criteria (such as the removal of prochlorperazine in the STOPP criteria about its use with parkinsonism or the replacement of “hypnotic Z-drugs” by “zolpidem,” which is the only Z-drug available in Portugal).

Despite the extensive literature on inappropriate prescribing generated over the last decade, much remains to be done regarding its implementation in clinical practice. Thus, further studies to assess the relationship between mis/over/underuse of drugs and adverse events (as hospitalizations, falls, deaths) should be performed with depth, as soon as possible, including an analysis of inherent costs.

## Conclusion

PIMs and PPOs are highly prevalent in geriatric patients and, therefore, more proactive interventions are needed to improve this scenario. The drugs most frequently identified as PIMs were those belonging to the central nervous system group, while PPOs were associated with drugs acting in the musculoskeletal and cardiovascular systems. The most common overuses were associated with benzodiazepines, which are predictors of an increased risk of falls, particularly when used for longer than 4 weeks. Omissions were more frequently related to the lack of vitamin D supplements, calcium-vitamin D supplements, angiotensin-converting enzyme inhibitors, and antiplatelet agents. Female gender, hospital provenience, and the higher number of medications prescribed were related to a higher rate of PIMs, in contrast to cerebrovascular disease and Parkinson’s disease. PPOs were associated with CCI and a history of recent fractures, while Parkinson’s disease and a metastatic solid tumor appeared to be protective. The fact that three specific criteria represent almost half of the total PIMs and PPOs show that targeted interventions can substantially improve the appropriateness of medication. Further national investigation is required, as well as international studies, focusing on the relationship between PIMs/PPOs and clinically relevant adverse events in order to better explore its consequences on patients’ health and to realize its economic impact.

## Data Availability

The raw data supporting the conclusions of this article will be made available by the authors, without undue reservation.
